# Transnational evaluation of the Sympathy for Violent Radicalization Scale: Measuring population attitudes toward violent radicalization in two countries

**DOI:** 10.1177/13634615211000550

**Published:** 2021-05-14

**Authors:** Rochelle L. Frounfelker, Thomas Frissen, Diana Miconi, Jordan Lawson, Robert T. Brennan, Leen d’Haenens, Cécile Rousseau

**Affiliations:** 1 McGill University; 2 KU Leuven, Maastricht University; 3 McGill University; 4 Boston College; 5 Boston College; 6 KU Leuven; 7 McGill University

**Keywords:** psychometrics, sympathy for violent radicalization, transnational evaluation, youth

## Abstract

Countering violent radicalization is a priority in many countries, prompting research that assesses attitudes and beliefs about violent radicalization in the general population. The majority of violent radicalization assessments have been developed among specific populations, with limited investigation into the generalizability and cross-cultural applicability of measurement tools. A transcultural investigation raises questions about the implicit assumptions and norms that inform instrument development. This research examined the psychometric properties of the Sympathy for Violent Radicalization Scale (SyfoR), a measure developed for use with Pakistani and Bangladeshi immigrant groups in the UK, in two convenience samples of youth and young adults in North America and Western Europe. We investigated the factor structure, reliability, and construct validity of adapted versions of the SyfoR among convenience samples of youth and young adults living in Belgium (*N* = 2014) and in Quebec, Canada (*N* = 1364) via online surveys administered to students engaged in secondary and post-secondary education. Results indicate that, in both samples, a reduced, 8-item version of the SyfoR has a 3-factor structure with good model fit statistics using confirmatory factor analysis and good internal consistency reliability. More studies are needed to assess the appropriateness of the SyfoR for use in diverse contexts and among diverse populations. The potential usefulness and harmfulness of measures of violent radicalization should balance the benefits of obtaining local data with the risks associated with pathologizing social dissent.

## Introduction

Societies across the globe are affected by radicalization leading to violence, and countering violent radicalization (VR) is a political priority in many countries (NCSTRT, 2015). The global landscape of VR is changing rapidly, with VR affecting both the Global North and the Global South. In the Global South, the world’s deadliest VR-related attack in 2017 was carried out by a suicide bomber in Mogadishu, Somalia (Institute for Economics and Peace, 2019). In the Global North, in 2019, a gunman opened fire at two Christchurch mosques in New Zealand. Although the impact of VR in terms of mortality is greatest in the Middle East and Northern Africa, VR activity is on the rise in sub-Saharan Africa (Institute for Economics and Peace, 2019). VR is also increasingly relevant in Western Europe and North America; for instance, the year 2017 was the deadliest in terms of far-right activity in North America since 2002 (Institute for Economics and Peace, 2019).

Definitions of radicalization, VR, extremism, and terrorism often appear in literature and public discussions with unclear boundaries, and are at times depicted as synonymous with one another. A working definition of radicalization is:

an individual or collective (group) process whereby, usually in a situation of political polarisation, normal practices of dialogue, compromise and tolerance between political actors and groups with diverging interests are abandoned by one or both sides in a conflict dyad in favour of a growing commitment to engage in confrontational tactics of conflict-waging. These can include either (i) the use of (non-violent) pressure and coercion, (ii) various forms of political violence other than terrorism or (iii) acts of violent extremism in the form of terrorism and war crimes. The process is, on the side of rebel factions, generally accompanied by an ideological socialization away from mainstream or status quo-oriented positions towards more radical or extremist positions involving a dichotomous world view and the acceptance of an alternative focal point of political mobilization outside the dominant political order as the existing system is no longer recognized as appropriate or legitimate. (Schmid, 2013, p. 18)

There are conflicting and blurry definitions of VR at least in part because it is a complex phenomenon that varies in expression based on the unique social, cultural, and historical contexts of diverse societies (Borum, 2012). Doosje et al. (2016) outline a typology of extremist discourses that may support and engage in violence: right-wing, left-wing, religious, nationalist/separatist, and single-issue extremism motivated by a specific topic (for example, animal rights) as opposed to an overarching ideology. These discourses are not mutually exclusive—for instance, ISIL could be characterized as utilizing both nationalistic and religious discourse (Frissen & d’Haenens, 2017)—and manifest in different ways across the globe (Doosje et al., 2016). Despite this diversity, the unifying characteristic of VR is increasing legitimization of, support for, and in some cases engagement in violence as a means to reach a specific (social, political, religious) goal (Schmid, 2013). This definition of VR thus encompasses violence, including most terrorist activities, as well as hate crimes and incidents. An act of terrorism is defined as an act whose primary purpose is to incite terror amongst the members of a particular non-combatant community in order to achieve a political goal (Keller, 2004). Hate incidents include all forms of expression (oral and written) or action involving prejudice or bias based on race, ethnicity, nationality, religion, sexual orientation, gender identity, and disability, whereas hate crimes constitute a criminal offence based on the same prejudice or bias (Dick, 2009).

Historically, much scholarly information on vulnerability to VR was based on interviews or secondary data analysis of radicalized individuals who committed acts of violence (see, for example, Silke, 2008). In more recent years, there has been a shift to conduct VR research with individuals in the general population (Frounfelker et al., 2019; Ozer & Bertelsen, 2018; Pedersen et al., 2018; Rousseau et al., 2020; Stankov et al., 2018). This is an important step forward to identify, at the population level, risk and protective factors for VR that include both majority and minority groups.

This research has focused on thoughts and beliefs that have often been conceptualized as precursors to violent action, proposing a continuum from the sympathy for those who engage in VR acts to the intention to commit violence (Borum, 2012). The overly simplistic linear associations frequently made between VR attitudes and violent behaviors has led to critiques that this body of research is used to legitimize securitization policies toward countering VR, including initiatives to screen or detect potentially violent individuals (Kundnani, 2014; Morgan, 2018; Ragazzi, 2016; Rousseau et al., 2017). Positive attitudes toward VR should not be considered an early indicator of risk of involvement in extremist and violent activities (Bartlett & Miller, 2012). Instead, evidence from the field of violence prevention suggests that the relationship between attitudes and behaviors is more complex: population-wide attitudes toward legitimizing some forms of violence may fuel or minimize the emergence of extremist groups, and provide a narrative to channel despair and rage in vulnerable individuals (Eisenman & Flavahan, 2017). Programs that utilize a public health approach that is informed by research on social determinants of health are promising alternatives to security-driven initiatives and could inform social and VR prevention policies at a broader level, taking into account the complexity of the phenomenon. Such programs distinguish between primary, secondary, and tertiary prevention efforts (Eisenman & Flavahan, 2017). Primary prevention targets the determinants of violent radical opinions and attitudes in the general population; secondary and tertiary prevention focus on vulnerable individuals that are attracted by extremist views or are already involved in extremist actions, alone or within a group. A clear distinction between these levels of intervention is crucial to a public health approach to VR and is reflected in empirical research that separates the study of positive attitudes toward VR in an effort to inform primary prevention within the general population from studies focusing on at-risk groups and/or individuals to inform secondary and/or tertiary prevention initiatives. Thus, understanding the determinants of these attitudes is an important starting point for primary prevention efforts against VR, such as school-based programming that emphasizes positive civic engagement, global citizenship, and constructive dialogue on polarizing topics (UNESCO, 2017).

### Transcultural psychiatry and assessing violent radicalization

In terms of measuring social constructs, transcultural psychiatry has paid attention not only to the extent to which psychosocial assessment tools are developed and tested in a variety of settings, but also to the ways in which instruments, just like diagnostic categories, may represent or perpetuate the imposition of hegemonic categories (Cartwright, 1851; Obeyesekere, 1985). This implies that, beyond the question of cross-cultural applicability of assessments and validity of measures among different populations, their underlying assumptions may need to be critiqued and challenged (Kirmayer, 2012). Within the context of the politically charged field of VR, how researchers understand and measure the construct of attitudes toward VR (and interpret the results of studies that use these measures) warrants close analysis to avoid an ideological instrumentalization of epidemiology and science.

In the field of VR, there is interest in identifying or developing scales that measure a “generic” construct of attitudes toward VR that can be used with different social and cultural groups (Ozer & Bertelsen, 2018). Although this is certainly an important goal if we wish to make valid inferences about VR attitudes across diverse populations, it is still unknown whether such a universal concept of VR exists or if, given the complexity of the phenomenon, local adaptations that tap into unique cultural and historical contexts and expressions of attitudes toward VR are necessary. A systematic review of instruments designed to assess attitudes, opinions, and readiness to engage in acts of radicalization and extremism indicates that the majority of existing assessments were developed and piloted for use with specialized populations on the basis of factors such as religion and involvement in violent action (Scarcella et al., 2016). Of 18 assessments identified by Scarcella et al. (2016), that were intended for research purposes, a total of six were developed with convicted terrorists or active militants, four were specifically designed for Muslim populations, and three were developed for individuals actively engaged in religious studies or places of worship. This indicates that scale development has been shaped by the prevailing assumptions that religious radicalization is the greatest threat, and that Muslims, and in particular devoted Muslims, are a high-risk group for VR (Younis & Jadhav, 2019). To our knowledge, to date, there has been little to no work validating and/or adapting these scales for use in the general population.

In 2014, researchers developed the Sympathy for Violent Radicalization Scale (SyfoR) to measure the level of endorsement of violent actions by others (Bhui et al., 2014b). This measure was developed in the UK, with a particular interest in Muslim immigrant communities. Details on the development of the SyfoR can be found elsewhere and included consulting with both Muslim and non-Muslim researchers on how to best measure radicalization and conducting focus groups with Muslim individuals with expertise in mental health, social science, and/or public health to identify indicators of sympathy for violent radicalization (Bhui et al., 2014b). A final 16-item scale of radicalization was piloted in English and was designed to ask respondents about sympathies for, or condemnation of, 16 different actions that fall within a range of behaviors engaged in by extremists (Bhui et al., 2014b). Researchers identified a 4-factor model, with factors labeled Radicalization, Defensive violence, Going to another country to fight British troops, and Sending British troops to another country. The 11 items comprising the first two factors (Radicalization and Defensive violence) were used as the outcome in studies investigating risk and protective factors for sympathy for VR among immigrant Muslim Pakistani and Bangladeshi adults in the UK (Bhui et al., 2014a, 2014b). Of importance, given that the SyfoR was originally developed and validated with a population-based representative sample of Bangladeshi and Pakistani immigrants in the UK to investigate positive attitudes toward radicalization at a primary prevention level, scores on the scale speak to population-wide attitudes, rather than individual risk of engaging in specific violent behaviors at a secondary or tertiary prevention level.

### Context of violent radicalization in Canada and Belgium

Since the early 2000s, concern over VR has grown in Europe, and in Belgium more specifically (Schmid, 2013). Belgium has the highest number of foreign terrorist fighters volunteering in Syria per capita in Europe (Neumann, 2015). Simultaneously, Belgium has witnessed an upsurge in right-wing extremism and (neo-)Nazism (Pano, 2018). As a consequence, the topic of VR is a priority for policy makers, national security services, and scholars alike. In Belgium, the national security services announced a fundamental reorganization in the coming years (known as ‘plan VSSE2021’) and for the year 2018 it received a historically high budget (Bové, 2018). In addition, Belgium has developed a more comprehensive counterterrorism approach, focusing on more prevention of radicalization measures (Coolsaet & Renard, 2018).

In autumn 2014, two attacks by lone actors in St-Jean (Québec) and Ottawa (Ontario) brought VR under the spotlight in Canada. Subsequently, the departure of youth to join ISIL (the Islamic State of Iraq and the Levant) in 2015 and the deadly attack against a mosque in Quebec in 2017 highlighted the increasing attraction exerted by different extremist discourses (extreme right and religious) in Quebec youth. In 2016, the Quebec government launched a national plan of action to prevent the upsurge in radicalization leading to violence, mobilizing the health and the education sectors through a series of measures (Gouvernement du Quebec, 2017).

### Objectives

One component of VR work being conducted in Belgium and Canada, as well as other countries, includes evaluating population-level trends in sympathy for VR. Researchers engaging in separate and distinct research on VR in both countries used adapted versions of the SyfoR as part of larger research projects on sympathy for VR among youth and young adults enrolled in secondary and post-secondary education.

The overall objectives of this study are to leverage a secondary data analysis of SyfoR data from both of these studies to (1) evaluate the psychometric properties of the SyfoR scale among diverse samples of youth and young adults; and (2) initiate a reflection around some of the implicit assumptions underlying scales for VR and their application in diverse contexts and among diverse populations. The specific aims of this study were to assess SyfoR factor structure, reliability, and validity in adapted versions used in Belgium and Quebec. We hypothesized that the factor structure of the SyfoR in Belgium and Quebec would be consistent with the factor structure of the original scale used in the UK (Bhui et al., 2014b). We also hypothesized that the scale would have good internal consistency reliability, as measured by Cronbach’s alpha. In addition, we hypothesized that scores on the SyfoR would be positively correlated with scores on a second measure of VR, the Radical Intention Scale (RIS).

## Methods

### Participants

#### Belgium

Participants included students at 38 secondary schools (including a school for adult education) in Flanders and the Brussels Capital Region from 2017 to 2018. We recruited a total of 2,218 young adults between 16 and 30 years old for a larger study examining the relationship between sociodemographic and psychosocial characteristics and sympathy for VR. Response rate was over 95%. Only respondents that completed the SyfoR scale in Dutch are included in this analysis (*n* = 2014).

#### Quebec

College students from 12 colleges in Quebec, Canada participated in a parent study on sympathy for violent radicalization from 2016 to 2017. Participants were eligible to participate if they were registered as full-time students in one of the participating colleges. Response rate varied greatly between the colleges, ranging from 2 to 19%. Only respondents between ages 16 and 24 who completed the SyfoR scale in French and had complete data on the SyfoR and Radical Intention Scales are included in this analysis (*n* = 1364).

### Measures

#### Sociodemographic characteristics

In both countries, participants self-reported sex, age, and current religious affiliation. Sex is measured as a binary variable (female/male); religion is a categorical variable and included the following options: None, Buddhist, Christian, Hindu, Jewish, Muslim, and Other. In Quebec, age was measured as a categorical variable with options including ages 16–18, 19–21, and 22–24. In Belgium, age was measured as a continuous variable; for purposes of comparison with the Quebec sample, this was transformed into a categorical variable including the age groups 16–18, 19–21, and 22–30.

#### Sympathy for Violent Radicalization Scale (SyfoR)

The SyfoR scale begins with the statement, “To what extent do you approve or reject the following behaviors?” Participants are then asked to document their level of support for different violent behaviors, such as the use of violence in the context of political protest and committing terrorist acts.

Researchers in each setting independently adapted the SyfoR for their own study. Researchers in both Belgium and Quebec dropped four items specific to British activity against VR, as those items were not relevant to the Canadian or Belgium context. That resulted in a final scale used in Belgium containing 12 questions. In Quebec, the final scale contained only nine of these 12 questions. Three questions related to extreme acts of violence, including threatening to commit terrorist acts, committing terrorist acts, and use of suicide bombs to fight injustice, were removed per the request of the ethics committee.

In Quebec, the SyfoR scale was measured on a seven-point Likert scale (1 = *I disagree completely* to 7 = *I agree completely*) consistent with the original version of the scale developed by Bhui and colleagues (2014b). Belgian researchers opted for a five-point Likert-scale (1 = *Total disapproval* to 5 = *Total approval*). Research indicates that data characteristics remain comparable between five- and seven-point Likert scales (Dawes, 2008). Higher scores indicate greater sympathy for VR. The final French and Dutch versions of the scale can be found in Appendices A and B.

#### Radicalism Intention Scale

The Radicalism Intention Scale (RIS) is a 4-item subscale of the Activism and Radicalism Intention Scales (ARIS) developed and validated by Moskalenko and McCauley (2009). The RIS assesses an individual’s readiness to support and participate in illegal and violent behavior in the name of one’s group or organization. Respondents rate their agreement to four statements: (1) “I would continue to support an organization that fights for my group’s political and legal rights even if the organization sometimes breaks the law”; (2) “I would continue to support an organization that fights for my group’s political and legal rights even if the organization sometimes resorts to violence”; (3) “I would participate in a public protest against oppression of my group even if I thought the protest might turn violent”; and (4) “I would attack police or security forces if I saw them beating members of my group.” The scale was developed and validated among general population samples of young adults in both the United States and Ukraine, and has been used by researchers in a variety of contexts with different populations and exhibits good psychometric properties (Ellis et al., 2016; Moyano & Trujillo, 2014). Participants in Belgium rated their response on a five-point Likert scale (1 = *Total disapproval* to 5 = *Total approval*), while in Quebec participants rated their response on a seven-point Likert scale (1 = *I disagree completely* to 7 = *I agree completely*), as originally intended by the authors of the scale. Higher total scores indicate more support for VR. Cronbach’s alpha for the RIS was .79 in the Quebec sample, and .79 in the Belgium sample. French and Dutch versions of the scale can be found in Appendices C and D.

### Procedures

In both Belgium and Quebec, data came from participants enrolled in larger studies on the topic of social polarization and radicalization previously conducted by the authors. In Canada, the study protocol and procedures were approved by the Ethics Committee of the Centre Integré Universitaire de Santé et de Services Sociaux du Centre-Ouest-de-l’Île-de-Montreal (CIUSSS-CODIM), protocol #2017-606,16-258. In addition, the research ethics board of each of the 12 colleges gave approval prior to data collection. In Belgium, the research ethics board of KU Leuven approved the study, protocol #G-2015 12 403. Standardized assessments were forward- and back-translated from English to French (Quebec) and from English to Dutch (Belgium; Van Ommeren et al., 1999).

#### Belgium

Researchers administered the survey as an online questionnaire in schools. Participants were provided with a written briefing, stating the purpose and nature of the study. To avoid priming respondents and biasing data, the briefing simply stated that the survey was about their everyday lives and their thoughts about contemporary society. Additionally, the briefing statement accentuated that participants’ anonymity was guaranteed and that they could discontinue at any time they wanted. Active informed consent was obtained before respondents could start the survey.

#### Quebec

Researchers uploaded the questionnaire on an intranet portal used by colleges to communicate with students and it remained online for a month. The project was described as a research study on adaptation to the current social context in Quebec. Students were informed that their involvement was voluntary and that their responses would be confidential. They consented to be part of the study via a consent form on the first page of the survey. Participants were able to discontinue the survey at any time. Contact information of research team and ethics board members was made available so they could answer any questions or concerns regarding the study.

### Data analysis

Analysis of Belgium and Quebec data was done in tandem and included the following steps. First, we used univariate descriptive statistics to describe the samples and individual distributions of the responses on the SyfoR. Polychoric correlations were computed and served as the basis for factor extraction analysis, as this approach leads to more accurate and robust estimation of associations than raw data (Holgado-Tello et al., 2010). An exploratory factor analysis (EFA) was performed on the reduced polychoric correlation matrix data to identify factor structure. We used maximum likelihood and determined factors by assessing eigenvalues and visually inspecting scree plots. Criteria for a factor were: a latent construct with at least two indicators, an eigenvalue greater than 0 (given we were using matrix data), and appearance on the scree plot to the left of the “elbow” (DeVellis, 2012). Given our large sample size, our choice of a latent construct having a minimum of two indicators was based on guidelines presented by Marsh et al. (1998). We rotated our model using oblique rotation. If individual items loaded onto a factor at or above .30, it was interpreted as indicating the item belonged within that factor. If an item loaded onto more than one factor at .30, it was allocated to the factor with the higher loading.

After extracting factors, confirmatory factor analysis (CFA) was performed on the raw data using maximum likelihood with a Satorra-Bentler estimation to adjust for the non-normality of the data (Satorra & Bentler, 1994). We used a number of model fit indices, specifically the chi-square statistic, the root mean square error of approximation (RMSEA), the standardized root mean square residual (SRMR), and the comparative fit index (CFI), to determine how well the model fit the study data. Criteria for model fit were: *x*^2^ *p >* .05, RMSEA < .10, SRMR < .08, and CFI > .90 (Hu and Bentler, 1999; Kline, 2016). In order to assess internal consistency reliability, Cronbach’s alpha was calculated for the total SyfoR scale, as well as subscales. Internal consistency was considered satisfactory if Cronbach’s alpha was ≥ .70 (DeVellis, 2012). Internal consistency reliability for 2-item subscales was calculated with the Spearman-Brown coefficient because it is less susceptible to bias than Cronbach’s coefficient in the case of 2-item constructs (Eisinga et al., 2013). Specific to the Belgium analysis, confirmatory factor analysis and a test of internal consistency reliability were conducted on both the final 11-item SyfoR and the reduced 8-item SyfoR used in Quebec to facilitate comparisons between the two data sets. We estimated Spearman’s correlation coefficients between SyfoR and RIS theta ( θ ) values to assess validity.

Statistical analyses were conducted with STATA version 15 software (StataCorp, 2015). Polychoric correlations were obtained with the polychoric package (Kolenikov, 2018). The Classical Test Theory Functions (CTT) package in R (Willse, 2018) was used to calculate Spearman-Brown coefficients for 2-item factors. Additional analyses were conducted using the ltm package in R (Rizopoulos, 2017).

## Results

Characteristics of study participants can be found in Table 1. The Belgium sample was overall younger than the Quebec sample, with 88.0% of participants between the ages of 16 and 18, compared to only 47.0% in Quebec. In Belgium, there were close to equal numbers of male and female participants, and 314 (15.6%) students identifying as Muslim. In contrast, the majority of respondents in Quebec were female (71.3%). In both countries, the majority of participants self-identified either as Christian or as having no religious affiliation.

**Table 1.** Sociodemographic characteristics of study participants in Belgium and Canada (Quebec)

Belgium (*N*=2014)			Quebec (*N*=1364)*		
	*n*	(%)		*n*	(%)
Gender					
Female	969	(48.1)		969	(71.3)
Male	1045	(51.9)		390	(28.7)
Age					
16--18	1773	(88.0)		641	(47.0)
19--21	224	(11.1)		574	(42.1)
22--30	17	(.8)	22--24	149	(11.0)
Religion					
None	1153	(57.3)		853	(64.3)
Buddhist	15	(.7)		5	(.4)
Christian	458	(22.7)		403	(30.4)
Hindu	9	(.5)		0	(0)
Jewish	4	(.2)		0	(0)
Muslim	314	(15.6)		41	(3.1)
Other	61	(3.0)		24	(1.8)

*Numbers do not total 1364 for each variable because of missing data.

As expected, the item-level distribution of responses in both samples was considerably skewed (see Table 2). In the Quebec data, 89.2% of participants responded *I disagree completely* to the question on organizing radical terrorist groups without personally taking part. The second most skewed item was “use of bombs to fight injustice,” with 86.5% reporting that they completely disagreed with such activity. Responses in Belgium were most skewed for the three SyfoR items dropped in the Quebec study. A total of 78.2%, 75.5%, and 73.9% of participants responded *Total disapproval* to actions of committing terrorist acts, threatening to commit terrorist acts, and use of suicide bombs to fight injustice, respectively.

**Table 2.** Item level response distributions for SyfoR Scale in Belgium and Quebec

		Belgium (*N*=2014)			Quebec (*N*=1364)		
	Item	Response Option	*n*	(%)	Response Option	*n*	(%)
1	Take part in non-violent protest	Total disapproval	296	(14.7)	I disagree completely	51	(3.7)
		Disapproval	283	(13.1)	I disagree to some extent	18	(1.3)
		Neutral	693	(34.4)	I disagree a little	17	(1.3)
		Approval	399	(19.8)	I neither disagree nor agree	60	(4.4)
		Total approval	343	(17.0)	I agree a little	47	(3.5)
					I agree to some extent	212	(15.5)
					I agree completely	959	(70.3)
2	Commit minor crime	Total disapproval	567	(28.2)	I disagree completely	705	(51.7)
		Disapproval	703	(34.9)	I disagree to some extent	275	(20.2)
		Neutral	571	(28.4)	I disagree a little	150	(11.0)
		Approval	127	(6.3)	I neither disagree nor agree	73	(5.4)
		Total approval	46	(2.3)	I agree a little	53	(3.9)
					I agree to some extent	80	(5.9)
					I agree completely	28	(2.1)
3	Use violence	Total disapproval	804	(49.9)	I disagree completely	953	(69.9)
		Disapproval	633	(31.4)	I disagree to some extent	221	(16.2)
		Neutral	464	(23.0)	I disagree a little	78	(5.7)
		Approval	76	(3.8)	I neither disagree nor agree	45	(3.3)
		Total approval	37	(1.8)	I agree a little	21	(1.5)
					I agree to some extent	39	(2.9)
					I agree completely	7	(.5)
4	Threaten to commit terrorist acts	Total disapproval	1520	(75.5)			
		Disapproval	129	(6.4)			
		Neutral	295	(14.7)			
		Approval	40	(2.0)			
		Total approval	30	(1.5)			
5	Organise radical terrorist groups without personally taking part	Total disapproval	1124	(55.8)	I disagree completely	121	(89.2)
		Disapproval	396	(19.7)	I disagree to some extent	76	(5.6)
		Neutral	408	(20.3)	I disagree a little	26	(1.9)
		Approval	53	(2.6)	I neither disagree nor agree	22	(1.6)
		Total approval	33	(1.6)	I agree a little	6	(.4)
					I agree to some extent	13	(1.0)
					I agree completely	4	(.3)
6	Commit terrorist acts	Total disapproval	1575	(78.2)			
		Disapproval	85	(4.2)			
		Neutral	285	(14.2)			
		Approval	37	(1.8)			
		Total approval	32	(1.59)			
7	Use of bombs to fight injustice	Total disapproval	1223	(60.7)	I disagree completely	118	(86.5)
		Disapproval	273	(13.6)	I disagree to some extent	83	(6.1)
		Neutral	387	(19.2)	I disagree a little	32	(2.4)
		Approval	71	(3.5)	I neither disagree nor agree	34	(2.5)
		Total approval	60	(3.0)	I agree a little	13	(1.0)
					I agree to some extent	16	(1.2)
					I agree completely	6	(.4)
8	Use of suicide bombs to fight injustice	Total disapproval	1488	(73.9)			
		Disapproval	150	(7.5)			
		Neutral	307	(15.2)			
		Approval	41	(2.0)			
		Total approval	28	(1.4)			
9	Violence to protect family	Total disapproval	165	(8.2)	I disagree completely	230	(16.9)
		Disapproval	183	(9.1)	I disagree to some extent	297	(21.8)
		Neutral	795	(34.5)	I disagree a little	126	(9.2)
		Approval	574	(28.5)	I neither disagree nor agree	164	(12.0)
		Total approval	397	(19.7)	I agree a little	146	(10.7)
					I agree to some extent	298	(21.9)
					I agree completely	103	(7.6)
10	Violence by organized groups to protect own race/religious group	Total disapproval	484	(24.0)	I disagree completely	536	(39.3)
		Disapproval	466	(23.1)	I disagree to some extent	309	(22.7)
		Neutral	750	(37.2)	I disagree a little	130	(9.5)
		Approval	235	(11.7)	I neither disagree nor agree	143	(10.5)
		Total approval	79	(3.9)	I agree a little	99	(7.3)
					I agree to some extent	124	(9.1)
					I agree completely	23	(1.7)
11	Violence to fight police injustice	Total disapproval	505	(25.1)	I disagree completely	609	(44.7)
		Disapproval	501	(24.9)	I disagree to some extent	257	(18.8)
		Neutral	680	(33.8)	I disagree a little	130	(9.5)
		Approval	215	(10.7)	I neither disagree nor agree	140	(10.3)
		Total approval	113	(5.6)	I agree a little	98	(7.2)
					I agree to some extent	84	(6.2)
					I agree completely	46	(3.4)
12	Violence to fight government injustice	Total disapproval	536	(26.6)	I disagree completely	634	(46.5)
		Disapproval	509	(25.3)	I disagree to some extent	254	(18.6)
		Neutral	673	(33.4)	I disagree a little	130	(9.5)
		Approval	199	(9.9)	I neither disagree nor agree	129	(9.5)
		Total approval	97	(4.8)	I agree a little	80	(5.9)
					I agree to some extent	94	(6.9)
					I agree completely	43	(3.2)

The 12-item SyfoR scale used in Belgium had a factor structure that differed from the same 12 items used in the UK. Item one, “Taking part in non-violent protest,” was removed before further analysis because it loaded on a single-item factor, the item was poorly correlated with other scale items (see Table 3), it had an item-rest correlation of .16, and the internal consistency reliability of the entire scale improved from .88 to .90 when it was removed. Of the remaining 11 items, four factors were retained, with questions number two and three, “Committing a minor crime” and “Use of violence” in political protest, loading onto their own factor. We labeled this factor Violent Protest. Two additional factors were labeled Defensive Violence and Injustice (items nine and ten, and items 11 and 12, respectively). All other items loaded onto a factor labeled Radicalization. CFA was conducted on the 4-factor model; the model met desired fit statistic cut-off scores for only one out of the four criteria, the CFI, and the 11-item scale was excluded from further analysis (see Table 4).

**Table 3.** Polychoric Correlation Matrices of 9 and 12 item Sympathy for Violent Radicalization Scale in Belgium (N=2014) and Quebec (N=1364)

Belgium (N=2014)											
	1	2	3	4	5	6	7	8	9	10	11
Syfor2	.29										
Syfor3	.17	.77									
Syfor4	−.08	.56	.70								
Syfor5	−.04	.65	.81	.78							
Syfor6	−.13	.56	.70	.94	.80						
Syfor7	.06	.55	.65	.78	.68	.78					
Syfor8	−.08	.55	.68	.91	.76	.91	.82				
Syfor9	.25	.29	.27	−.08	.13	−.10	.16	−.08			
Syfor10	.19	.51	.56	.43	.54	.44	.53	.44	.55		
Syfor11	.25	.51	.60	.48	.49	.45	.57	.46	.49	.66	
Syfor12	.28	.56	.64	.48	.50	.47	.61	.50	.45	.67	.88

Quebec (N=1364)											
Syfor2	.25										
Syfor3	.16	.77									
Syfor4											
Syfor5	−.06	.66	.82								
Syfor6											
Syfor7	.08	.59	.75		.78						
Syfor8											
Syfor9	.26	.37	.49		.44		.45				
Syfor10	.22	.43	.58		.59		.58		.74		
Syfor11	.26	.54	.72		.58		.72		.58	.67	
Syfor12	.24	.55	.74		.61		.75		.57	.67	.92

**Table 4.** Estimated standardized factor loadings and model fit statistics for 8 and 11 item SyfoR using CFA in Belgium (N=2014) and Quebec (N=1364)

	Belgium (*N*=2014) (11-item)				Belgium (*N*=2014) (8-item)			Quebec (*N*=1364) (8-item)		
	Violent Protest	Radicalization	Defensive Violence	Injustice	Radicalization	Defensive Violence	Injustice	Radicalization	Defensive Violence	Injustice
Syfor2	.76	--	--	--	.75	--	--	.68	--	--
Syfor3	.92	--	--	--	.91	--	--	.90	--	--
Syfor4	--	.94	--	--	--	--	--	--	--	--
Syfor5	--	.70	--	--	.78	--	--	.71	--	--
Syfor6	--	.93	--	--	--	--	--	--	--	--
Syfor7	--	.71	--	--	.65	--	--	.70	--	--
Syfor8	--	.91	--	--	--	--	--	--	--	--
Syfor9	--	--	.56	--	--	.55	--	--	.74	--
Syfor10	--	--	.86	--	--	.88	--	--	.90	--
Syfor11	--	--	--	.91	--	--	.91	--	--	.94
Syfor12	--	--	--	.93	--	--	.93	--	--	.94
χ ^2^	χ ^2^ (38) = 827.02, *p*< .05				χ ^2^ (17) = 288.53, *p*< .05			χ ^2^ (17) = 149.56, *p*< .05		
RMSEA	.102				.089			.076		
CFI	.923				.958			.962		
SRMR	.091				.049			.037		

In the Quebec sample, EFA with an oblique rotation on the 9-item SyfoR indicated a 5-factor model. Item one, “Taking part in non-violent protest,” was again dropped from the analysis as it loaded separately onto its own factor, was poorly correlated with other scale items (see Table 3), and had an item-rest correlation of .15. Removal of the item improved internal consistency reliability of the scale from .85 to .87. Of the remaining four factors, one contained only a single scale item that loaded at .30 or higher, question number two, “Committing a minor crime.” In addition to the factor failing to meet our criteria of having at least two items, question number two loaded more strongly onto one of the other three factors (.42 compared to .35). Researchers moved forward with a final, 3-factor model for further analysis.

In the Belgian sample, using the same nine items, the EFA with oblique rotation also indicated a 5-factor model. Aligned with Quebec, we dropped item one from the scale based on the same criteria as in the Quebec analysis: the item loaded separately onto its own factor, it was poorly correlated with other scale items, and it had an item-rest correlation of .22. Additionally, the internal consistency reliability of the scale improved from .85 to .87 after its removal. Of the remaining four factors, one had no items loading ≥.3 and was subsequently removed. Thus, in alignment with the Quebec data, researchers moved forward with a 3-factor model for further analysis, with factors labeled Radicalization (items two, three, five, and seven), Defensive Violence (items nine and ten), and Injustice (items 11 and 12).

CFA was conducted on the 3-factor model with the remaining 8-item scale in the Quebec and Belgium data, and both samples had adequate fit statistics, except the chi-square, which was attributed to sample size (see Table 4). In Quebec, Cronbach’s alpha for the entire scale was .87; the Cronbach’s alpha for the Radicalization factor was .80, also meeting cut-off criteria of greater than .70. Spearman-Brown coefficients yielded a satisfactory internal reliability for both the defensive violence factor .88 and .97 for the Injustice factor. In Belgium, Cronbach’s alpha for the entire 8-item scale was .87; Cronbach’s alpha for the Radicalization factor was .85. The Spearman coefficient was .78 for Defensive Violence and .95 for Injustice. All SyfoR subscales were associated with the RIS at p < .05. The factor Radicalization had the strongest correlation with the RIS in both Quebec (.55) and Belgium (.59) (see Table 5).

**Table 5.** Spearman correlation coefficients between SyfoR factor theta values and RIS theta values in Belgium (N=2014) and Quebec (N=1364)*

Belgium	SyfoR ‘Defensive Violence’	SyfoR ‘Radicalization’	SyfoR ‘Injustice’
SyfoR ‘Injustice’	.49	.59	
Radical Intention Scale	.27	.59	.47
Quebec			
SyfoR ‘Radicalization’	.47		
SyfoR ‘Injustice’	.64	.61	
Radical Intention Scale	.41	.55	.51

*All correlations were significant at *p* < .05

## Discussion

This study assessed the psychometric properties of adapted versions of the Sympathy for Violent Radicalization Scale used in Canada (Quebec) and Belgium (Flanders and Brussels Capital Region). In both Quebec and Belgium, the 8-item SyfoR had a 3-factor structure, with good model fit statistics using CFA and good internal consistency reliability. Each of the three factors was positively correlated with the RIS, with the strongest correlation between the Radicalization factor and RIS theta scores. The factor structure of the 11-item SyfoR in Belgium did not replicate the findings of Bhui and colleagues (2014b), and had poor model fit statistics.

There are various explanations for the lack of alignment in findings on the psychometric properties of the SyfoR used in Belgium and Quebec and the original scale developed and used in the UK. Perhaps most obviously, there were considerable adaptations, including the removal of some items (in Quebec) and modification of the Likert-scale response categories (in Belgium) from the original scale that may have had an impact on factor structure. The validation of the scale in the UK was conducted with a representative population sample of Pakistani and Bangladeshi immigrants between the ages of 18 and 45 via quota sampling recruitment and in-person interviews (Bhui et al., 2014b); the studies in both Quebec and Belgium were conducted among convenience samples of youth and young adults engaged in secondary and post-secondary education via an online portal. Additionally, the data analysis approaches used were not identical. In the UK, authors used principal component factor analysis with an orthogonal rotation to identify factors (Bhui et al., 2014b); in this study, we used exploratory factor analysis and confirmatory factor analysis within a structural equation framework to assess factor structure and overall model fit. These differences limit the assertions that can be made about the overall consistency (or lack thereof) of the psychometric properties of the scale, its cross-cultural validity, and the generalizability of the assessment to different settings.

Our results suggest avenues of reflection on the construct of VR attitudes. First, the fact that SyfoR item one, “support for non-violent protest,” loaded separately onto its own factor contradicts the idea of a continuum among different forms of transgression of social norms. It clearly illustrates that not all forms of political protest should be considered as indicators of sympathy for VR, and that their inclusion in scales measuring this concept may lead to a problematic amalgamation between social protest, legitimate dissent, and VR. The inclusion of this item in a scale related to VR implies that the refusal of the social status quo is a first step in extremism; this is more a political and ideological position than an evidence-based fact. Nonviolent protest may on the contrary constitute an alternative avenue to challenge and overcome other grievances. Including some of these alternative avenues in scales assessing attitudes toward radicalization (violent and nonviolent) would be very pertinent if conceptually these constructs were identified as distinct. Rigorous fine-grained measures of attitudes toward VR that are able to differentiate between violent and non-violent radicalization are needed.

Second, compared to the original version of the scale, our analyses, even if on a reduced number of items, indicated the presence of a 3-factor rather than a 2-factor solution, suggesting a distinction between defensive violence to protect one’s group (e.g., family) and violence to protest against social and political injustice. This raises an important, and yet unresolved debate. On one hand, the idea of self-defense, which includes family members, is almost universally accepted as a legitimate form of violence, and its inclusion as a possibly problematic attitude can be strongly questioned. On the other hand, in recent VR-related attacks, the defense of a close group has been repeatedly put forward as a legitimate justification for mass killing, as was the case in the Pittsburgh synagogue event. Thus, there is a need to further investigate feelings of personal, familial, and group threat to see how they are constructed and when they are fueled by hate discourses or related to actual objective threats. These results support an ecological understanding of positive attitudes toward VR as a multi-faceted phenomenon determined by the interaction of personal, social, and political factors (Eisenman & Flavahan, 2017). Future studies should delve further into this distinction to investigate how different components of attitudes are related to specific risk and protective factors to better inform prevention and intervention.

### Limitations

This study has important limitations. First, the use of convenience samples and the low response rate in Quebec caution the generalizability of findings to other populations because of selection bias. The reliability of the scale was assessed solely with Cronbach’s alpha and Spearman-Brown coefficient, as our study design did not allow for assessment of test-retest or inter-rater reliability. Additionally, we were only able to assess validity of the SyfoR in relationship to youth self-report on the RIS. Also, although we make comparisons between the 8-item SyfoR results in Belgium and Quebec, these should be interpreted cautiously, as the scales used different Likert-scale response options. Finally, the original SyfoR scale and the Belgian and Quebec adaptations were developed in Western societies, and results cannot be generalized to other settings (including low- and middle-income countries).

## Conclusion

Measurement of perceptions, attitudes, and intentions around VR should theoretically be undertaken with assessments developed and evaluated using best practices. Given the heated socio-political context surrounding the issue of VR, in particular the tension between a security agenda and the protection of civil rights, instruments should be critiqued in terms of their potential usefulness to document social polarization versus their potential to inappropriately legitimize the profiling and pathologizing of social dissent. This study provides an initial step in assessing the reliability and validity of the SyfoR when adapted for use in a population of young students. It also raises questions in terms of the normative frames used to classify attitudes toward VR as positive or negative, and invites readers to delve into the complexity of social attitudes in times of polarization. It is not yet clear if the SyfoR has cross-cultural validity and generalizability to normative populations of youth and young adults. More formative research needs to be done before encouraging researchers and policy makers to make standardized use of the assessment.

## Supplemental Material

sj-pdf-1-tps-10.1177_13634615211000550 - Supplemental material for Transnational evaluation of the Sympathy for Violent Radicalization Scale: Measuring population attitudes toward violent radicalization in two countries Click here for additional data file. Supplemental material, sj-pdf-1-tps-10.1177_13634615211000550 for Transnational evaluation of the Sympathy for Violent Radicalization Scale: Measuring population attitudes toward violent radicalization in two countries by Rochelle L. Frounfelker, Thomas Frissen, Diana Miconi, Jordan Lawson, Robert T. Brennan, Leen d’Haenens and Cécile Rousseau in Transcultural Psychiatry

sj-pdf-2-tps-10.1177_13634615211000550 - Supplemental material for Transnational evaluation of the Sympathy for Violent Radicalization Scale: Measuring population attitudes toward violent radicalization in two countries Click here for additional data file. Supplemental material, sj-pdf-2-tps-10.1177_13634615211000550 for Transnational evaluation of the Sympathy for Violent Radicalization Scale: Measuring population attitudes toward violent radicalization in two countries by Rochelle L. Frounfelker, Thomas Frissen, Diana Miconi, Jordan Lawson, Robert T. Brennan, Leen d’Haenens and Cécile Rousseau in Transcultural Psychiatry

sj-pdf-3-tps-10.1177_13634615211000550 - Supplemental material for Transnational evaluation of the Sympathy for Violent Radicalization Scale: Measuring population attitudes toward violent radicalization in two countries Click here for additional data file. Supplemental material, sj-pdf-3-tps-10.1177_13634615211000550 for Transnational evaluation of the Sympathy for Violent Radicalization Scale: Measuring population attitudes toward violent radicalization in two countries by Rochelle L. Frounfelker, Thomas Frissen, Diana Miconi, Jordan Lawson, Robert T. Brennan, Leen d’Haenens and Cécile Rousseau in Transcultural Psychiatry

sj-pdf-4-tps-10.1177_13634615211000550 - Supplemental material for Transnational evaluation of the Sympathy for Violent Radicalization Scale: Measuring population attitudes toward violent radicalization in two countries Click here for additional data file. Supplemental material, sj-pdf-4-tps-10.1177_13634615211000550 for Transnational evaluation of the Sympathy for Violent Radicalization Scale: Measuring population attitudes toward violent radicalization in two countries by Rochelle L. Frounfelker, Thomas Frissen, Diana Miconi, Jordan Lawson, Robert T. Brennan, Leen d’Haenens and Cécile Rousseau in Transcultural Psychiatry
