# Supplementary material for: Transnational evaluation of the Sympathy for Violent Radicalization Scale: Measuring population attitudes toward violent radicalization in two countries
Source: Transcult Psychiatry. 2021 May 14;58(5):669–82. doi: 10.1177/13634615211000550 (PMC8733345; doi:10.1177/13634615211000550)
Supplement: sj-pdf-1-tps-10.1177_13634615211000550 - Supplemental material for Transnational evaluation of the Sympathy for Violent Radicalization Scale: Measuring population attitudes toward violent radicalization in two countries [file sj-pdf-1-tps-10.1177_13634615211000550.pdf]

Appendix A. Sympathy for Violent Radicalization Scale used in Quebec Study

Dans quelle mesure êtes-vous en accord ou en désaccord avec les gens qui commettent les actions suivantes:

1. Complètement en désaccord
2. En désaccord dans une certaine mesure
3. Un peu en désaccord
4. Ni en accord ni en désaccord
5. Un peu en accord
6. En accord dans une certaine mesure
7. Complètement en accord
8. Ne sais pas
9. Refuse/préfère ne pas répondre

participent à des manifestations non-violentes	1	2	3	4	5	6	7	8	9
commettent des délits mineurs lors de manifestations politiques (e.g. dommages à la propriété)	1	2	3	4	5	6	7	8	9
ont recours à la violence lors de manifestations politiques	1	2	3	4	5	6	7	8	9
font l'organisation de groupes radicaux violents	1	2	3	4	5	6	7	8	9
ont recours à la violence pour protéger leur famille	1	2	3	4	5	6	7	8	9
le recours à la violence par des groupes organisés pour protéger les gens de leur propre groupe	1	2	3	4	5	6	7	8	9
ont recours à la violence pour se battre contre l'injustice de la police	1	2	3	4	5	6	7	8	9
ont recours à la violence pour se battre contre l'injustice du gouvernement	1	2	3	4	5	6	7	8	9
utilisent des armes/bombes pour se battre contre les injustices	1	2	3	4	5	6	7	8	9
