# Supplementary material for: Transnational evaluation of the Sympathy for Violent Radicalization Scale: Measuring population attitudes toward violent radicalization in two countries
Source: Transcult Psychiatry. 2021 May 14;58(5):669–82. doi: 10.1177/13634615211000550 (PMC8733345; doi:10.1177/13634615211000550)
Supplement: sj-pdf-2-tps-10.1177_13634615211000550 - Supplemental material for Transnational evaluation of the Sympathy for Violent Radicalization Scale: Measuring population attitudes toward violent radicalization in two countries [file sj-pdf-2-tps-10.1177_13634615211000550.pdf]

Appendix B. Sympathy for Violent Radicalization Scale used in Belgium Study

Sympathies for Radicalization, Dutch

QUESTION: In welke mate keur je volgende vormen van gedrag goed of af?

- 5 point Likert:
- Totale afkeuring, Afkeuring, Neutraal, Goedkeuring, Totale goedkeuring

SyfoR 1:	Deelnemen aan vreedzaam politiek protest.
SyfoR 2:	De wet overtreden uit politiek protest (vb. vandalisme)
SyfoR 3:	Geweld gebruiken bij politiek protest
SyfoR 4:	Dreigen een terroristische aanslag te plegen
SyfoR 5:	Radicale gewelddadige groeperingen vormen
SyfoR 6:	Een terroristische aanslag uitvoeren
SyfoR 7:	Gebruik van wapens of bommen om onrechtvaardigheid te bestrijden
SyfoR 8:	Gebruik van zelfmoordbommen om onrecht te bestrijden
SyfoR 9:	Geweld gebruiken om je familie te beschermen
SyfoR 10:	Gebruik van geweld door georganiseerde groeperingen om hun mensen te beschermen
SyfoR 11:	Geweld gebruiken tegen onrechtvaardigheid door de politie
SyfoR 12:	Geweld gebruiken tegen onrechtvaardigheid door de regering
