# Supplementary material for: Transnational evaluation of the Sympathy for Violent Radicalization Scale: Measuring population attitudes toward violent radicalization in two countries
Source: Transcult Psychiatry. 2021 May 14;58(5):669–82. doi: 10.1177/13634615211000550 (PMC8733345; doi:10.1177/13634615211000550)
Supplement: sj-pdf-3-tps-10.1177_13634615211000550 - Supplemental material for Transnational evaluation of the Sympathy for Violent Radicalization Scale: Measuring population attitudes toward violent radicalization in two countries [file sj-pdf-3-tps-10.1177_13634615211000550.pdf]

Appendix C. Radical Intention Scale used in Quebec study

1. Je continuerais à soutenir une organisation qui se bat pour les droits politiques et légaux de mon groupe, même si parfois cette organisation enfreint la loi.

1	2	3	4	5	6	7
complètement en désaccord						complètement d’accord

2. Je continuerais à soutenir une organisation qui se bat pour les droits politiques et légaux de mon groupe même si parfois l’organisation a recours à la violence.

1	2	3	4	5	6	7
complètement en désaccord						complètement d’accord

3. Je participerais à une manifestation publique contre l’oppression de mon groupe même si je pensais que la manifestation pourrait devenir violente.

1	2	3	4	5	6	7
complètement en désaccord						complètement d’accord

4. J’attaquerais la police ou les forces de l’ordre si je les voyais battre des membres de mon groupe.

1	2	3	4	5	6	7
complètement en désaccord						complètement d’accord
