# Supplementary material for: Transnational evaluation of the Sympathy for Violent Radicalization Scale: Measuring population attitudes toward violent radicalization in two countries
Source: Transcult Psychiatry. 2021 May 14;58(5):669–82. doi: 10.1177/13634615211000550 (PMC8733345; doi:10.1177/13634615211000550)
Supplement: sj-pdf-4-tps-10.1177_13634615211000550 - Supplemental material for Transnational evaluation of the Sympathy for Violent Radicalization Scale: Measuring population attitudes toward violent radicalization in two countries [file sj-pdf-4-tps-10.1177_13634615211000550.pdf]

Appendix D. Radical Intention Scale used in Belgium study

QUESTION: In welke mate keur je volgende vormen van gedrag goed of af?

- 5 point Likert:
- Totale afkeuring, Afkeuring, Neutraal, Goedkeuring, Totale goedkeuring

RIS 1:	Het steunen van een organisatie die de rechten van mijn groep/cultuur verdedigt, zelfs al overtreedt die organisatie nu en dan de wet
RIS 2:	Het steunen van een organisatie die de rechten van mijn groep / cultuur verdedigt, zelfs als die organisatie daarvoor geweld gebruikt
RIS 3:	Deelnemen aan gewelddadig protest omdat mijn groep / cultuur onderdrukt wordt.
RIS4:	Politie of veiligheidsdiensten aanvallen als ze geweld gebruiken tegen leden van mijn groep / cultuur.
